# Successful Resolution of Glucose Toxicity With the Use of Fixed-Ratio Combination Injection of Basal Insulin and Short-Acting Glucagon-Like Peptide 1 (GLP-1) Receptor Agonist

**DOI:** 10.7759/cureus.25889

**Published:** 2022-06-13

**Authors:** Hiroshi Miura, Naokazu Muramae, Kenta Mori, Kazunori Otsui, Kazuhiko Sakaguchi

**Affiliations:** 1 General Internal Medicine, Kobe University Hospital, Kobe, JPN; 2 General Internal Medicine, Kobe University Graduate School of Medicine, Kobe, JPN

**Keywords:** cgm, basal insulin, short-acting glp-1 ra, glucose toxicity, fixed ratio of combination injection therapy

## Abstract

Chronic hyperglycemia leads to a decrease in glucose-stimulated insulin secretion and an increase in insulin resistance. Resolving these glucose toxicities is pivotal in type 2 diabetes therapy because the decline in insulin secretion and insulin sensitivity causes further hyperglycemia. Conventionally, multiple daily insulin injection therapy was applied in such a situation. However, it could not be easily introduced, especially in outpatients. We present a case involving the successful resolution of glucose toxicity easily, immediately, and safely by using a fixed-ratio combination (FRC) injection of basal insulin and short-acting glucagon-like peptide 1 (GLP-1) receptor agonists (GLP-1 RA). Additionally, we discuss the advantages of this new injection therapy.

## Introduction

Basal insulin and glucagon-like peptide 1 (GLP-1) receptor agonists (GLP-1 RAs) have complementary modes of action [1,2]. Basal insulin reduces fasting plasma glucose levels by suppressing hepatic glucose production [3]. GLP-1 RAs exert their hypoglycemic effects by slowing gastric motility, augmenting glucose-stimulated insulin secretion from pancreatic β cells, and reducing glucagon secretion from pancreatic α cells [4]. Although GLP-1 RAs also reduce appetite and body weight, nausea or occasional vomiting are common adverse events of this injection therapy. Fixed-ratio combination (FRC) therapy involves a single injection containing a mixture of basal insulin and GLP-1 RA in a fixed ratio once daily [5]. Clinical studies have shown that FRC therapy improved both fasting and postprandial glucose levels compared to basal insulin or GLP-1 RA injection therapy in isolation [6,7]. Moreover, hypoglycemic risks, body weight gain, and gastrointestinal adverse effects were lower compared to basal insulin or GLP-1 RA injection therapy alone [6,7].

Among GLP-1 RAs, only the short-acting variant has manifested the ability to delay gastric emptying, which remarkably stabilizes postprandial glucose levels. In contrast, augmenting glucose-stimulated insulin secretion, another hypoglycemic effect of GLP-1 RAs, depends on the endogenous insulin secretion capacity of each patient [8], and delaying gastric emptying does not depend on the delay [9,10].

Glucose toxicity refers to a decrease in glucose-stimulated insulin secretion and an increase in insulin resistance in the target organ due to chronic hyperglycemia [11]. Since the decline in insulin secretion and insulin sensitivity causes further hyperglycemia, cutting this vicious cycle and recovering the patients' insulin secretion capacity and insulin sensitivity is the main objective of type 2 diabetes therapy. Insulin therapy, especially the basal-bolus method (multiple daily injection therapy), has been conventionally used to reliably and promptly resolve glucose toxicity. However, basal-bolus insulin therapy is complicated and accompanied by hypoglycemic and obesogenic effects [1]. Due to its safer and simpler nature compared to the basal-bolus insulin injection therapy, FRC, especially that containing short-acting GLP-1 RA, is expected to stabilize fasting and postprandial glucose levels even when insulin secretion capacity is reduced due to glucose toxicity.

As proof of concept, we present a case where we administered an FRC preparation named SOLIQUA® [IGlarLixi: basal insulin, insulin glargine (1 U/10 μL), short-acting GLP-1 RA, and lixisenatide (1 μg/10 μL); Japanese original mixing ratio; Sanofi, Paris, France] to a patient with poor glycemic control, efficiently resolving glucose toxicity in a short period of time in an outpatient setting.

## Case presentation

A 59-year-old woman with type 2 diabetes mellitus was referred to our hospital due to progressive bodyweight loss (10 kg in six months). After her diagnosis of diabetes two years earlier, she had been receiving treatment with oral hypoglycemic agents (OHAs). Her appetite was not affected, but she reported that the coronavirus disease 2019 (COVID-19) pandemic had forced her to stop her habitual exercise routine. Her family doctor had prescribed empagliflozin 10 mg, pioglitazone 10 mg, and sitagliptin 50 mg. Since empagliflozin had been started two years earlier, it was unlikely that this medication was the reason for her weight loss in the last six months. She also reported a recent increase in urination at night.

The patient's height, body weight, and body mass index (BMI) on the day of referral (day one) were 157.0 cm, 70 kg, and 28.4 kg/m^2^, respectively. Her vitals were stable, and she had no central nervous system symptoms suggestive of hyperglycemic hyperosmolar syndrome. In addition, she did not have any apparent diabetic macro- or microvascular complications. The laboratory data on the day of referral are shown in Table 1.

**Table 1:** Laboratory data on admission AER: albumin excretion rate; WBC: white blood cell; RBC: red blood cell; Hb: hemoglobin; Plt: platelet; Alb: albumin; T-Bil: total bilirubin; ALT: alanine aminotransferase; AST: aspartate aminotransferase; ChE: choline esterase; LD: lactate dehydrogenase; BUN: blood urea nitrogen; Cre: creatinine; eGFR: estimated glomerular filtration rate; T-Chol: total cholesterol; HDL-Chol: high-density lipoprotein cholesterol; TG: triglyceride; Glu: glucose; HbA1c: hemoglobin A1c; GA: glycoalbumin; CPR: C-peptide immunoreactivity; TSH: thyroid-stimulating hormone; f-T4: free thyroxine

Test	Result	Units	Reference
Urinalysis			
Specific gravity	1.046		(1.006-1.030)
Protein	(-)		
Glucose	(4+)		
Ketone	(-)		
AER	10.2	mg/g/Cr	(<30)
Peripheral blood			
WBC	8.3	× 10^3^/μL	(4.0-8.0)
RBC	5.66	× 10^6^/μL	(3.86-4.92)
Hb	16.9	g/dL	(11.6-14.8)
Plt	343	× 10^3^/μL	(158-348)
Blood chemistry			
Total protein	7.6	mg/dL	(6.5-8.0)
Alb	4.9	mg/dL	(3.8-5.2)
T-Bil	0.9	mg/dL	(0.2-1.0)
ALT	44	IU/L	(6-43)
AST	66	IU/L	(11-33)
γ-GTP	39	IU/L	(10-50)
ChE	489	IU/L	(201-421)
LD	177	IU/L	(120-245)
BUN	8.6	mg/dL	(9-21)
Cre	0.71	mg/dL	(0.2-0.9)
eGFR	64.7	mL/min/1.73m^2^	
T-Chol	190	mg/dL	(130-220)
HDL-Chol	49	mg/dL	(40-65)
TG	257	mg/dL	(50-150)
Glu	187	mg/dL	(70-110)
HbA1c	9.3	%	(4.6-6.2)
GA	24.3	%	(11-16)
CPR	3.76	ng/mL	(1.2-2.0)
TSH	2.56	μU/mL	(0.34-3.5)
f-T4	1.18	ng/dL	(0.9-1.8)

**Figure 1:**
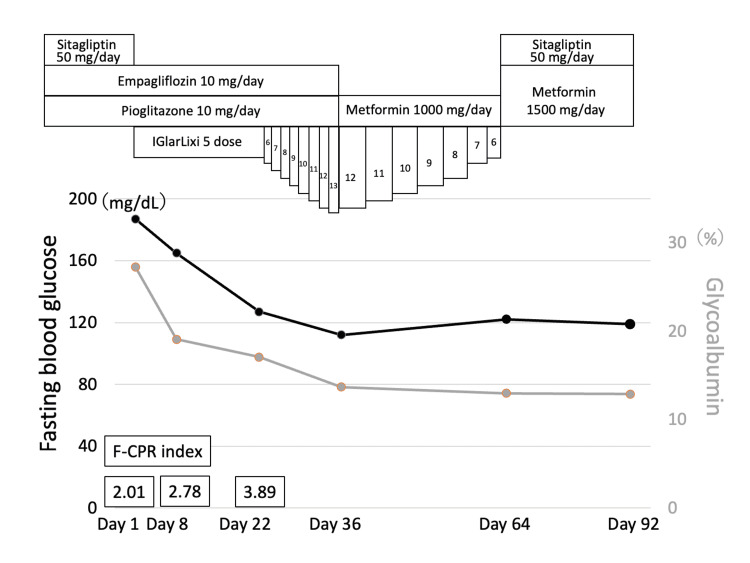
Clinical course after the initiation of IGlarLixi F-CPR index was calculated as (fasting CPR level/fasting plasma glucose level) × 100 CPR: C-peptide immunoreactivity

Her thyroid function was normal. There was no evidence of any other diseases that may have caused her bodyweight loss. Since her HbA1c and fasting plasma glucose levels were 9.3% and 187 mg/dL, respectively, on the day of referral, her body weight loss was assumed to be due to poor glycemic control. Urinary ketones were absent. Her C-peptide response (CPR) index (CPI) [(fasting CPR level/fasting plasma glucose level) × 100] was 2.01. Her rapid weight loss suggested an increase in catabolism, and insulin therapy was required to resolve her glucose toxicity. As she did not want to be hospitalized, a consensus on choosing FRC for outpatient treatment was reached. Figure 1 illustrates the patient's clinical course.

**Figure 2:**
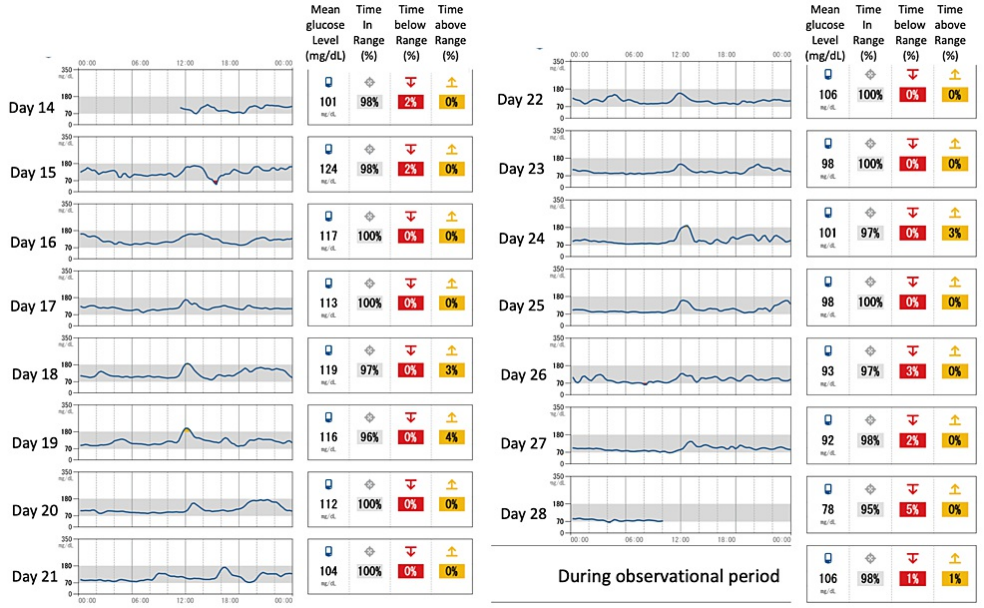
Daily data of continuous glucose monitoring Time in range: the percentage of time with 70-180 mg/dL of glucose (target glucose range of type 2 diabetic patient); time below range: the percentage of time with below 70 mg/dL; time above range: the percentage of time with above 180 mg/dL

**Figure 3:**
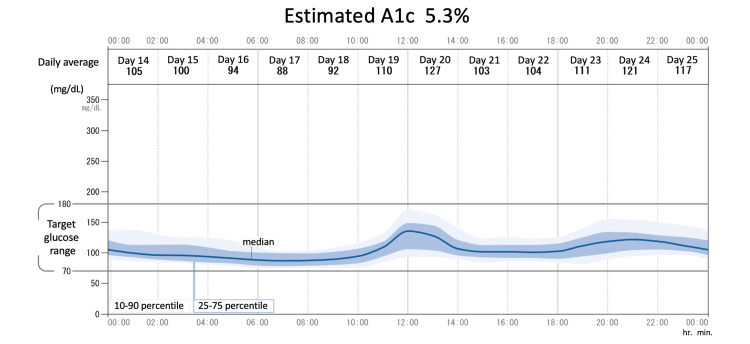
Ambulatory glucose profile report The patient's glucose profile for two weeks is shown with the median line, 25th to 75th percentile line, and 10th to 90th percentile with the estimated HbA1c level. The average daily glucose level is also demonstrated

At the time of initiation of FRC, the patient's empagliflozin and pioglitazone were continued, and sitagliptin was discontinued. Five doses of IGlarLixi (each dose of IGlarLixi contains 1 U of IGlar and 1 μg of lixisenatide) were started to be administered subcutaneously daily prebreakfast without self-monitoring blood glucose (SMBG) tests. After initiating FRC therapy without dietary guidance, her body weight increased to 77 kg in a week. Her fasting plasma glucose level, glycoalbumin level, and fasting CPI were 165 mg/dL, 19.1%, and 2.78, respectively, after one week of treatment (day eight) with five doses of IGlarLixi. We confirmed that the reason for her weight loss was increasing catabolism; hence, we provided her dietary guidance (1700 kcal/day) and instructed her to continue IGlarLixi at the same dose during the next week along with measuring fasting blood glucose level by SMBG. Her fasting plasma glucose level, glycoalbumin level, and fasting CPI were 127 mg/dL, 17.1%, and 3.89, respectively, on day 15. In the third week after initiating IGlarLixi, the upward-self-titrating method was selected to increase the dose of IGlarLixi by one dose if the fasting glucose level remained over 110 mg/dL for two consecutive days. A professional continuous glucose monitoring (CGM) (FreeStyle Libre Pro; Abbott Diabetes Care, Alameda, CA) was worn from day 14 to day 28. Her blood glucose level almost completely normalized without significant hypoglycemia (Figure 2), and her estimated HbA1c was 5.3% on CGM (Figure 3).

During this visit, she complained of pollakiuria. Therefore, her oral medication was changed from empagliflozin and pioglitazone to metformin 1000 mg/day. Also, the downward self-titration was selected to reduce the dose of IGlarLixi by one dose if the fasting glucose level remained below 130 mg/dL for two consecutive days. IGlarLixi administration was discontinued on day 42. After successfully resolving glucose toxicity and improving her endogenous insulin secretion capacity, the patient was referred back to her family doctor with a prescription of sitagliptin 50 mg and metformin 1500 mg as oral antidiabetic medications. The patient weighed 76 kg at the last visit.

## Discussion

CPI is known as an indicator of endogenous insulin secretion capacity [12,13]. We presented a case where once-daily FRC injection therapy safely and rapidly improved glycemic control and successfully resolved glucose toxicity to restore endogenous insulin secretion capacity in a female patient, eventually leading to the withdrawal of injection therapy.

Resolving glucose toxicity is crucial in type 2 diabetes patients with poor glycemic control because early intervention and strict glycemic control of blood glucose lead to improved insulin secretion capacity of pancreatic β cells along with a decrease in insulin resistance in insulin-target organs [14,15]. Needless to say, insulin therapy with appropriate rehydration and correction of abnormalities in electrolyte balance should be applied in severe metabolic disorders such as diabetic ketoacidosis or hyperglycemic hyperosmolar syndrome. However, even in the absence of such severe metabolic disorders, insulin therapy has often been administered to patients with suboptimal glycemic control to resolve glucose toxicity promptly. Moreover, it has been reported that insulin therapy maintains β cell function better than OHA (sulfonylurea or metformin) therapy in clinical trials [16]. Additionally, because continuous subcutaneous insulin infusion (CSII) therapy has shown more favorable conservation of β cell function than basal-bolus insulin therapy, strict glycemic control in fasting and postprandial time seems important [16]. However, CSII and basal-bolus insulin therapies are complicated to introduce, especially for outpatients.

GLP-1 RAs are classified into short-acting and long-acting types according to the duration of their pharmacological action [4]. The main difference between the two types involves their effect on gastric emptying [4]. The long-acting type loses the ability to slow gastric motility because of tachyphylaxis [17]. Moreover, reduced glucose-stimulated insulin secretion (glucose toxicity for insulin secretion) is thought to be caused by the downregulation of the GLP-1 receptor on the surface of β cells [18]. Therefore, long-acting GLP-1 RA cannot exert its hypoglycemic effect completely during glucose toxicity. Thus, IGlarLixi (SOLIQUA®) seemed suitable for resolving glucose toxicity.

Sodium-glucose cotransporter-2 (SGLT-2) inhibitor is another unique medication that improves glycemic control in glucose toxicity and conserves β cell function [19]. As this drug exerts a hypoglycemic effect, increasing urinary glucose excretion without stimulating insulin release leads to a decrease in hypoglycemic risk. However, diabetic ketoacidosis has been reported when it was used in patients with poor insulin secretion capacity [20].

## Conclusions

Even when the glucose-stimulated insulin secretion of β cells is impaired, good glycemic control can be obtained by using FRC therapy, especially that involving short-acting GLP-1 RA, as seen in our patient. A once-daily injection of IGlarLixi has the potential to replace basal-bolus insulin therapy in patients with type 2 diabetes with poor glycemic control. However, it has yet to be deciphered as to which therapy among FRC and basal-bolus insulin conserves β cell function for a long span. A randomized controlled prospective trial is required to address this question.
